# Suicidal ideation in primary care patients suffering from panic disorder with or without agoraphobia

**DOI:** 10.1186/s12888-018-1894-5

**Published:** 2018-09-24

**Authors:** Tobias Teismann, Karoline Lukaschek, Thomas S. Hiller, Jörg Breitbart, Christian Brettschneider, Ulrike Schumacher, Jürgen Margraf, Jochen Gensichen, the Jena Paradies Study Group

**Affiliations:** 1 Mental Health Research and Treatment Center, Ruhr-Universität Bochum, Massenbergstraße 11, 44787 Bochum, Germany; 2 Institute of General Practice and Family Medicine, University Hospital of Ludwig-Maximilians- University Munich, Pettenkoferstr. 10, D-80336 Munich, Germany; 3 Institute of Epidemiology, Helmholtz Zentrum München, German Research Center for Environmental Health, Ingolstädter Landstraße 1, D-85764 Neuherberg, Germany; 4 Institute of General Practice and Family Medicine, Jena University Hospital, Friedrich-Schiller-Universität, Bachstr. 18, D-07743 Jena, Germany; 5 Department of Health Economics and Health Services Research, Hamburg Center for Health Economics, University Medical Center Hamburg-Eppendorf, Martinistr. 52, D-20246 Hamburg, Germany; 6 Centre for Clinical Studies, Jena University Hospital, Salvador-Allende-Platz 27, D-07747 Jena, Germany

**Keywords:** Suicidal ideation, Panic disorder, Agoraphobia, Primary care

## Abstract

**Background:**

Suicidal ideation is common in patients suffering from panic disorder. The present study investigated rates of suicidal ideation and risk factors for suicidal ideation in a sample of primary care patients suffering from panic disorder with or without agoraphobia.

**Methods:**

A total of *N* = 296 patients [*n* = 215 (72.6%) women; age: *M* = 43.99, *SD* = 13.44] were investigated. Anxiety severity, anxiety symptoms, avoidance behavior, comorbid depression diagnosis, severity of depression, age, sex, employment status, living situation and frequency of visits at the general practitioner were considered as risk factors of suicidal ideation.

**Results:**

Suicidal ideation was experienced by 25% of the respondents. In a logistic regression analysis, depression diagnosis and depression severity emerged as significant risk factors for suicidal ideation. Anxiety measures were not associated with suicidal ideation.

**Conclusion:**

Suicidal ideation is common in primary care patients suffering from panic disorder with or without agoraphobia. Individuals with greater burden of mental illness in terms of mood disorder comorbidity and depressive symptomatology are especially likely to suffer from suicidal ideation.

## Background

Suicidal ideation is highly prevalent in the general population (8.5%; [1]) and even more in clinical samples (18–32%; [2]). Thinking about suicide is a risk factor for completed suicide [3], and even passive ideation, such as a wish to die, has been identified as a predictor for death by suicide [4]. Since there is a rapid transition from suicidal ideation to plans and attempts [5] early detection of suicidal ideation is essential to initiate timely interventions. A better knowledge of risk factors for suicidal ideation, especially in primary care patients, may therefore help to identify potentially suicidal patients.

One of the strongest risk factors for suicidal ideation and behavior is the presence of a mental disorder, most prominently affective disorders, schizophrenia and substance use disorders [6], but also anxiety disorders [7]. Among anxiety disorders, panic disorder has received a great deal of attention: Using data from the National Institute of Mental Health Epidemiologic Catchment Area (ECA) study, Weissman et al. [8] found that 47% of individuals with panic disorder reported lifetime suicidal ideation. In European community studies lower - but still significant - rates of lifetime suicidal ideation have been found in persons suffering from panic disorder (23.7%: [2]; 15.7%: [9]). In studies on treatment-seeking patients suffering from panic disorder 31% reported having had suicidal thoughts in the past year [10] and 25% in the past week [11] - even after excluding patients suffering from comorbid major depression. Taken together, there is strong evidence that patients suffering from panic disorder display heightened risk for suicidal ideation, which underscores the debilitating character of the disorder. Yet, most information concerning suicidal ideation in panic disorder stems from studies on the general population or psychiatric services [7] and may not apply to primary care settings.

The prevalence of anxiety disorders is higher in primary care settings than in the general population [12] and current panic disorder/agoraphobia diagnoses are found in about 4% of primary care patients [13]. Mental health care for panic disorder/agoraphobia is sought and obtained mostly from general practitioners [14]. Yet, suicidal ideation often goes unrecognized in these settings [15], even though almost 50% of patients who died through suicide had seen their general practitioner in the month preceding their death [16] and up to 20% visit a primary care provider within 1 day of their death [17]. In light of these findings, primary care settings have been emphasized as an essential component of effective and comprehensive suicide prevention [18].

Therefore, the first aim of this study was to identify rates of suicidal ideation in primary care patients diagnosed with panic disorder with or without agoraphobia. The second aim was to identify characteristic symptoms that are associated with suicidal ideation and may therefore guide risk assessment in primary care patients. Anxiety severity, anxiety symptoms, agoraphobic avoidance as well as depression diagnosis, depression severity, demographic markers (age, sex, living alone, unemployment) previously shown to be associated with suicidal ideation [19], and frequency of visits at the general practitioner, as an alternative measure of general distress, were considered as potential predictors of suicidal ideation.

## Methods

### Study design

A cross-sectional study that was nested in a randomized trial comparing a practice team-supported, self-managed exposure program for patients with panic disorder (PD) with or without agoraphobia (AG) in general practices to usual care was conducted [Trial Registration: ISCRTN64669297]. The study was approved by the Ethics Committee of the Friedrich- Schiller-University at the Medical Faculty (Jena, Germany). Details of the study design and recruiting process have been published previously [20]. To be eligible for the trial, patients had to meet the following inclusion criteria: (1) being at least 18 years of age, (2) being diagnosed with PD/AG (ICD-10: F41.0 or F40.01) by a general practioner-led clinical interview, (3) showing a minimum total score on the ‘Overall Anxiety and Impairment Scale’ (OASIS) of 8 points [21] and at least two positive answers on the panic module of the ‘Patient Health Questionnaire’ (PHQ; [22]) at the time of inclusion, (4) having sufficient German language skills, (5) having a telephone connection, (5) being able of giving written informed consent to participate in the study. Patients were excluded if they met one or more of the following exclusion criteria according to general practitioner’s clinical evaluation: suffering from suicidal tendencies in need of immediate treatment, acute or chronic psychosis, dependence on psychoactive substance(s), or severe physical illness, being pregnant, receiving professional psychotherapeutic treatment for their anxiety disorder at the time of inclusion. Of note, none of the patients screened for the study was excluded due to suicidal tendencies in need of immediate treatment. Data collection occurred between August 2012 and October 2014.

### Participants

At baseline, a sample of 419 patients took part in an exhaustive assessment. The full set of questionnaires was completed by 296 patients [*n* = 215 (72.6%) women; age: *M* = 43.99, *SD* = 13.44] and only patients who completed all questionnaires were included in the current analysis. Patients with complete data and patients who completed only a subset of questionnaires did neither differ in sex nor diagnosis. However, patients who completed only a subset of questionnaires were older than patients with complete data, *t*(414) = 5.23, *p* < .000.

Half of the patients were married (*n* = 150; 50.6%), 33.4% were singles (*n* = 99), 11.8% were separated/divorced (*n* = 35) and 4.1% were widowed (*n* = 12). A minority of patients was unemployed (*n* = 41, 13.6%). The vast majority (*n* = 231, 78%) suffered from panic disorder with agoraphobia, and *n* = 65 (22%) from panic disorder without agoraphobia. Fifty-five individuals (18.6%) suffered from a comorbid depressive disorder. Most patients saw their general practitioner on a very regular basis, with *M* = 8.76 (*SD* = 8.72) visits within the last 6 months. All participants were Caucasian.

### Measures

#### Suicidal ideation

Suicidal ideation was measured using the respective item from the Patient Health Questionnaire’ (PHQ-9; [22]): “Over the last two weeks, how often have you been bothered by thoughts that you would be better of dead, or of hurting yourself in some way?” Response options range from “not at all”, “several days”, “more than half the days” to “nearly every day”. Response to the PHQ-9-suicide item has recently been shown to be a strong predictor of suicide attempt and suicide death within the following year [23].

#### Anxiety symptoms

Anxiety Symptoms were measured with the Beck Anxiety Inventory (BAI; [24]). The BAI is a 21-item self-report measure that surveys anxiety symptoms on a 4-point severity scale. It has demonstrated good internal consistency (Cronbach’s *α* ≥ .85 across clinical and non-clinical samples [25]). The internal consistency for the BAI in the current sample was *α* = .91.

#### Anxiety severity

The Overall Anxiety Severity and Impairment Scale (OASIS; [26]) is a 5-item self-report measure to assess frequency and intensity of anxiety symptoms, functional impairment related to these symptoms and behavioral avoidance. All items are answered on a 5-point scale ranging from 0 (not at all) to 4 (extreme). The OASIS has a unidimensional factor structure and has been shown to have a good internal consistency (Cronbach’s *α* = .89) as well as convergent and divergent validity [21]. Internal consistency was rather modest in the current sample: *α* = .67.

#### Agoraphobic avoidance

The ‘Mobility Inventory’ (MI; subscale ‘alone’; [27]) was used to assess agoraphobic avoidance behavior across a variety of different situations. All items are answered on a 5-point scale ranging from 1 (never) to 5 (always). The MI-total score has an excellent internal consistency: Cronbach’s *α* ≥ .94 [27]. Accordingly, internal consistency was good in the present sample: *α* = .95.

#### Depressive symptoms

Severity of depressive symptoms was measured by the ‘Patient Health Questionnaire’ (PHQ-[9, 22]). The PHQ-9 assesses the occurrence of nine depressive symptoms within the previous 2 weeks. It has been shown to have good internal consistency: Cronbach’s *α* ≥ .86 [28]. Internal consistency was *α* = .84 in the present sample. The suicide item was excluded from the PHQ-sum score to avoid overlapping and thus part-whole correlations.

### Statistical analyses

We conducted bivariate correlation analyses (Spearman) of all study variables. Then, a logistic regression analysis was performed with suicidal ideation (yes/no) as criterion. Age, sex, current unemployment (yes/no), living status (living alone: yes/no), frequency of visits at the general practitioner, diagnosis of panic disorder with vs. without agoraphobia, comorbid depression disorder (yes/no), depressive symptom severity, anxious symptoms, anxiety severity and avoidance behavior were considered as predictors of suicidal ideation. Derived odds rations (including 95% confidence interval, CI) are presented for each predictor variable. Tolerance in the collinearity statistics was higher than 0.2 for all models, indicating the absence of multicollinearity. All statistical analyses were conducted using the statistical analysis program IBM SPSS Statistics 21 (SPSS Inc., Illinois, Chicago).

## Results

### Descriptive statistics and correlations

Descriptive statistics for each measure are presented in Table 1. A quarter of the sample (25%; *n* = 74) reported suicidal ideation (i.e., scores > 0 on the PHQ-9 suicide item) within the last 2 weeks: Sixty-one patients (20.5%) indicated suffering from suicidal ideation for “several days” during the last 2 weeks, nine patients (3.1%) indicated that they had suffered from suicidal ideation for “more than half the days” and four patients (1.4%) indicated suffering from suicidal ideation “nearly every day”.

**Table 1:** Means, standard deviations and correlations of study variables

	*M* (*SD*)	PHQ-SI
PHQ-SI	0.31 (0.59)	–
BAI	28.71 (12.33)	.233**
OASIS	12.54 (2.66)	.190**
MI	2.28 (0.83)	.077
PHQ	11.10 (5.25)	.417**

*BAI* Beck Anxiety Inventory, *MI* Mobility Inventory, *OASIS* Overall Anxiety Severity and Impairment Scale, *PHQ* Patient Health Questionaire, *PHQ-SI* Patient Health Questionaire – Suicide Ideation Item

**= *p* < .01

As shown in Table 1, suicidal ideation correlates positively with anxiety symptoms, anxiety severity and depression. However, an association with avoidance behavior was not detected.

### Risk factors of suicidal ideation

Table 2 shows the results of the logistic regression analysis with suicidal ideation as criterion variable.

**Table 2:** Logistic regression analysis predicting suicidal ideation

	OR (95% CI)	*21p*
Age	1.01 (.99–1.04)	n.s.
Sex	.83 (.40–1.72)	n.s.
Living alone (yes/no)	1.20 (.64–2.61)	n.s.
Unemployment (yes/no)	.79 (.32–1.96)	n.s.
GP-visits	1.02 (.99–1.06)	n.s.
PD with AG vs. PD without AG	1.07 (.49–2.35)	n.s.
Comorbid depression (yes/no)	2.33 (1.12–4.84)	.023
PHQ	1.22 (1.13–1.32)	.000
BAI	1.01 (.98–1.04)	n.s.
OASIS	1.02 (.90–1.16)	n.s.
MI	.70 (.45–1.07)	n.s.

*AG* Agoraphobia, *BAI* Beck Anxiety Inventory, *MI* Mobility Inventory, *GP* general practitioner, *OASIS* Overall Anxiety Severity and Impairment Scale, *PD* Panic disorder, *PHQ* Patient Health Questionaire

Only comorbid depression disorder and depression severity predicted concurrent suicidal ideation. Neither anxiety symptoms nor anxiety severity and avoidance behavior emerged as significant predictors.

## Discussion

Results of the present study indicate that suicidal ideation is common in primary care patients suffering from panic disorder with or without agoraphobia. In total, 25% of the patients reported suicidal ideation within the last 2 weeks. This percentage closely approximates those from other studies on patient samples suffering from anxiety disorders [11, 29] and points to the debilitating character of the disorder. In a logistic regression analysis, only comorbid depression diagnosis and depression severity emerged as significant predictors of suicidal ideation. Anxiety measures were not associated with suicidal ideation. Furthermore, patients suffering from panic disorder with agoraphobia were not more likely to suffer from suicidal ideation than patients suffering from panic disorder without agoraphobia.

Taken together, the current findings complement previous research that found depression to be the key risk factor for suicidal ideation and behavior within samples of anxiety patients (e.g., [29, 30]). However, the relationship between panic disorder and suicidal ideation and behavior remains a controversial one, as some studies found such an association, even in the absence of coexisting major depression [8]. Furthermore, panic attacks have repeatedly been shown to be associated with imminent suicide risk [31, 32]. Against this background the findings of Yaseen et al. [32] are of particular interest: they report that only fear of dying within a panic attack is prospectively associated with suicide attempts, whereas no other panic attack symptom was associated with subsequent suicidal ideation and behavior.

In terms of clinical implications, the results of the current study highlight the importance of suicide risk assessments in anxiety patients. Though suicide assessments for individuals suffering from depression has received considerable attention within primary care (e.g., [33]), findings point to the additional need for routine suicide risk assessments in patients suffering from panic disorder [29]. Taken together, general practitioners should be encouraged to ask about suicidal ideation in their anxiety patients, particularly in those suffering from comorbid depression.

The strengths of the present investigation are its embedding within the framework of the thoroughly designed Jena-PARADIES study and the use of well-established depression and anxiety instruments. Yet, there are some limitations to the current study. First, the present investigation is a cross-sectional analysis; therefore, conclusions on causality cannot be drawn. Second, suicidal ideation was assessed using a single item self-report measure, instead of a multi-item self-report questionnaire. Single-item assessment are associated with an increased risk of misclassification [34]; at the same time, Simon et al. [23] reported strong evidence for the predictive ability of the PHQ suicide item [23]. Third, most patients reported that suicidal ideation would occur only occasionally. It therefore remains unclear to what extent results generalize to samples with more intensive suicidal thoughts. Finally, the group of predictors used in the study was far from exhaustive. In particular, we did not measure anxiety cognitions or anxiety sensitivity, both variables that have been associated with suicidal ideation [32, 35].

## Conclusion

The present study showed that suicidal ideation is not uncommon in primary care patients suffering from panic disorder with or without agoraphobia. Patients particularly affected are those suffering from comorbid mood disorders and depressive symptomatology [29].
